# Antibody cocktail to SARS-CoV-2 spike protein prevents rapid mutational escape seen with individual antibodies

**DOI:** 10.1126/science.abd0831

**Published:** 2020-06-15

**Authors:** Alina Baum, Benjamin O. Fulton, Elzbieta Wloga, Richard Copin, Kristen E. Pascal, Vincenzo Russo, Stephanie Giordano, Kathryn Lanza, Nicole Negron, Min Ni, Yi Wei, Gurinder S. Atwal, Andrew J. Murphy, Neil Stahl, George D. Yancopoulos, Christos A. Kyratsous

**Affiliations:** Regeneron Pharmaceuticals Inc., Tarrytown, NY 10591, USA.

## Abstract

Antibodies targeting the spike protein of SARS-CoV-2 present a promising approach to combat the COVID19 pandemic; however, concerns remain that mutations can yield antibody resistance. We investigate the development of resistance against four antibodies to the spike protein that potently neutralize SARS-CoV-2, individually as well as when combined into cocktails. These antibodies remain effective against spike variants that have arisen in the human population. However, novel spike mutants rapidly appeared following in vitro passaging in the presence of individual antibodies, resulting in loss of neutralization; such escape also occurred with combinations of antibodies binding diverse but overlapping regions of the spike protein. Importantly, escape mutants were not generated following treatment with a non-competing antibody cocktail.

A promising approach to combat the COVID19 pandemic involves development of antiviral antibodies targeting the spike protein of SARS-CoV-2. The spike protein is a key mediator of viral infectivity required for attachment and entry into target cells by binding the ACE2 receptor (*1*, *2*). A significant concern for any antiviral therapeutic is the potential for acquiring drug resistance due to the rapid mutation of viral pathogens. Such resistance becomes more obvious when selective pressure is applied in the setting of drug treatment. For example, when HIV drugs were initially used individually, such drug-selected mutations resulted in widespread resistance. The subsequent success of combination therapy for HIV demonstrated that requiring the virus to simultaneously mutate at multiple genetic positions may be the most effective way to avoid drug resistance.

We have recently described parallel efforts – utilizing genetically-humanized mice and B cells from convalescent humans – to generate a very large collection of highly-potent fully human neutralizing antibodies targeting the RBD of the spike protein of SARS-CoV-2 (*3*). The prospective goal of generating this very large collection was to select pairs of highly potent individual antibodies that could simultaneously bind the RBD spike, and thus might be ideal partners for a therapeutic antibody cocktail that could not only be an effective treatment, but might also protect against antibody resistance due to virus escape mutants that could arise in response to selective pressure from single antibody treatments.

To assess the efficacy of our recently described antiviral antibodies against the breadth of spike RBD variants represented in publicly available SARS-CoV-2 sequences identified through the end of March 2020 (representing over 7000 unique genomes), we used the VSV pseudoparticle system expressing the SARS-CoV-2 spike variants. Our top eight neutralizing antibodies maintained their potency against all tested variants (Table 1), demonstrating broad coverage against circulating SARS-CoV-2.

**Table 1:** Anti-SARS-CoV2 spike mAbs demonstrate broad neutralization across SARS-CoV-2 spike RBD variants. Eight anti-spike antibodies were tested against sixteen SARS-CoV-2 spike protein RBD variants identified from viral sequences circulating through end of March 2020. The listed variants were encoded into pVSV-SARS-CoV-2-S (mNeon) pseudoparticles and neutralization assays were performed in Vero cells. IC50(M) values are shown for each variant. There was no observed neutralization with hIgG1 isotype control (N/A).

	**Anti-SARS-CoV-2 spike monoclonal antibodies**								
**Variants**	**REGN10989**	**REGN10987**	**REGN10933**	**REGN10934**	**REGN10964**	**REGN10954**	**REGN10984**	**REGN10986**	**Isotype control**
Wild-type	7.23 × 10^–12^	4.06 × 10^–11^	4.28 × 10^–11^	5.44 × 10^–11^	5.70 × 10^–11^	9.22 × 10^–11^	9.73 × 10^–11^	9.91 × 10^–11^	N/A
Q321L	1.46 × 10^–11^	5.02 × 10^–11^	6.85 × 10^–11^	6.84 × 10^–11^	5.65 × 10^–11^	2.32 × 10^–10^	2.75 × 10^–10^	2.06 × 10^–10^	N/A
V341I	1.61 × 10^–11^	3.38 × 10^–11^	3.37 × 10^–11^	7.42 × 10^–11^	1.13 × 10^–10^	2.52 × 10^–10^	2.49 × 10^–10^	1.92 × 10^–10^	N/A
A348T	7.33 × 10^–12^	2.98 × 10^–11^	4.13 × 10^–11^	1.42 × 10^–10^	3.52 × 10^–11^	1.84 × 10^–10^	2.01 × 10^–10^	1.03 × 10^–10^	N/A
N354D	1.14 × 10^–11^	2.68 × 10^–11^	5.89 × 10^–11^	9.76 × 10^–11^	1.93 × 10^–10^	2.84 × 10^–10^	2.64 × 10^–10^	2.49 × 10^–10^	N/A
S359N	4.30 × 10^–12^	2.41 × 10^–11^	2.12 × 10^–11^	3.04 × 10^–11^	6.83 × 10^–11^	1.09 × 10^–10^	1.23 × 10^–10^	8.91 × 10^–11^	N/A
V367F	1.33 × 10^–11^	1.78 × 10^–11^	2.40 × 10^–11^	3.20 × 10^–11^	8.92 × 10^–11^	1.29 × 10^–10^	1.53 × 10^–10^	1.49 × 10^–10^	N/A
K378R	1.21 × 10^–11^	2.40 × 10^–11^	3.52 × 10^–11^	4.65 × 10^–11^	6.19 × 10^–11^	1.65 × 10^–10^	1.88 × 10^–10^	1.54 × 10^–10^	N/A
R408I	1.09 × 10^–11^	1.71 × 10^–11^	1.98 × 10^–11^	2.75 × 10^–11^	4.96 × 10^–11^	9.88 × 10^–11^	1.35 × 10^–10^	6.14 × 10^–11^	N/A
Q409E	2.12 × 10^–11^	4.06 × 10^–11^	5.65 × 10^–11^	5.94 × 10^–11^	6.61 × 10^–11^	2.64 × 10^–10^	1.52 × 10^–10^	1.95 × 10^–10^	N/A
A435S	1.10 × 10^–11^	3.88 × 10^–11^	4.71 × 10^–11^	8.07 × 10^–11^	7.90 × 10^–11^	2.11 × 10^–10^	2.18 × 10^–10^	1.51 × 10^–10^	N/A
K458R	7.51 × 10^–12^	1.68 × 10^–11^	3.43 × 10^–11^	3.46 × 10^–11^	5.46 × 10^–11^	1.45 × 10^–10^	1.59 × 10^–10^	1.00 × 10^–10^	N/A
I472V	2.27 × 10^–11^	4.18 × 10^–11^	9.17 × 10^–11^	9.40 × 10^–11^	1.01 × 10^–10^	3.44 × 10^–10^	2.61 × 10^–10^	2.24 × 10^–10^	N/A
G476S	6.80 × 10^–12^	1.86 × 10^–11^	1.41 × 10^–10^	3.51 × 10^–11^	3.42 × 10^–11^	1.83 × 10^–10^	2.10 × 10^–10^	1.13 × 10^–10^	N/A
V483A	8.78 × 10^–12^	2.60 × 10^–11^	1.54 × 10^–11^	4.43 × 10^–11^	4.50 × 10^–11^	1.12 × 10^–10^	1.71 × 10^–10^	9.70 × 10^–11^	N/A
Y508H	1.71 × 10^–11^	2.75 × 10^–11^	4.77 × 10^–11^	6.73 × 10^–11^	1.02 × 10^–10^	2.05 × 10^–10^	2.83 × 10^–10^	2.01 × 10^–10^	N/A
H519P	4.51 × 10^–12^	2.20 × 10^–11^	3.03 × 10^–11^	3.56 × 10^–11^	4.45 × 10^–11^	1.40 × 10^–10^	1.08 × 10^–10^	6.14 × 10^–11^	N/A

Next, escape mutants were selected under pressure of single antibodies, as well as of antibody combinations, by using a replicating VSV-SARS-CoV-2-S virus (Fig. 1A). We rapidly identified multiple independent escape mutants for each of the four individual antibodies within the first passage (Fig. 1, B and C, and Fig. 2). Some of these mutants became readily fixed in the population by the second passage, representing 100% of sequencing reads, and are resistant to antibody concentrations of up to 50ug/ml (~10,000-100,000 greater concentration than IC50 against parental virus). Sequencing of escape mutants (Fig. 2) revealed that single amino acid changes can ablate binding even to antibodies that were selected for breadth against all known RBD variants (Table 1), and that neutralize parental virus at low pM IC50 (*3*).

**Fig. 1:**
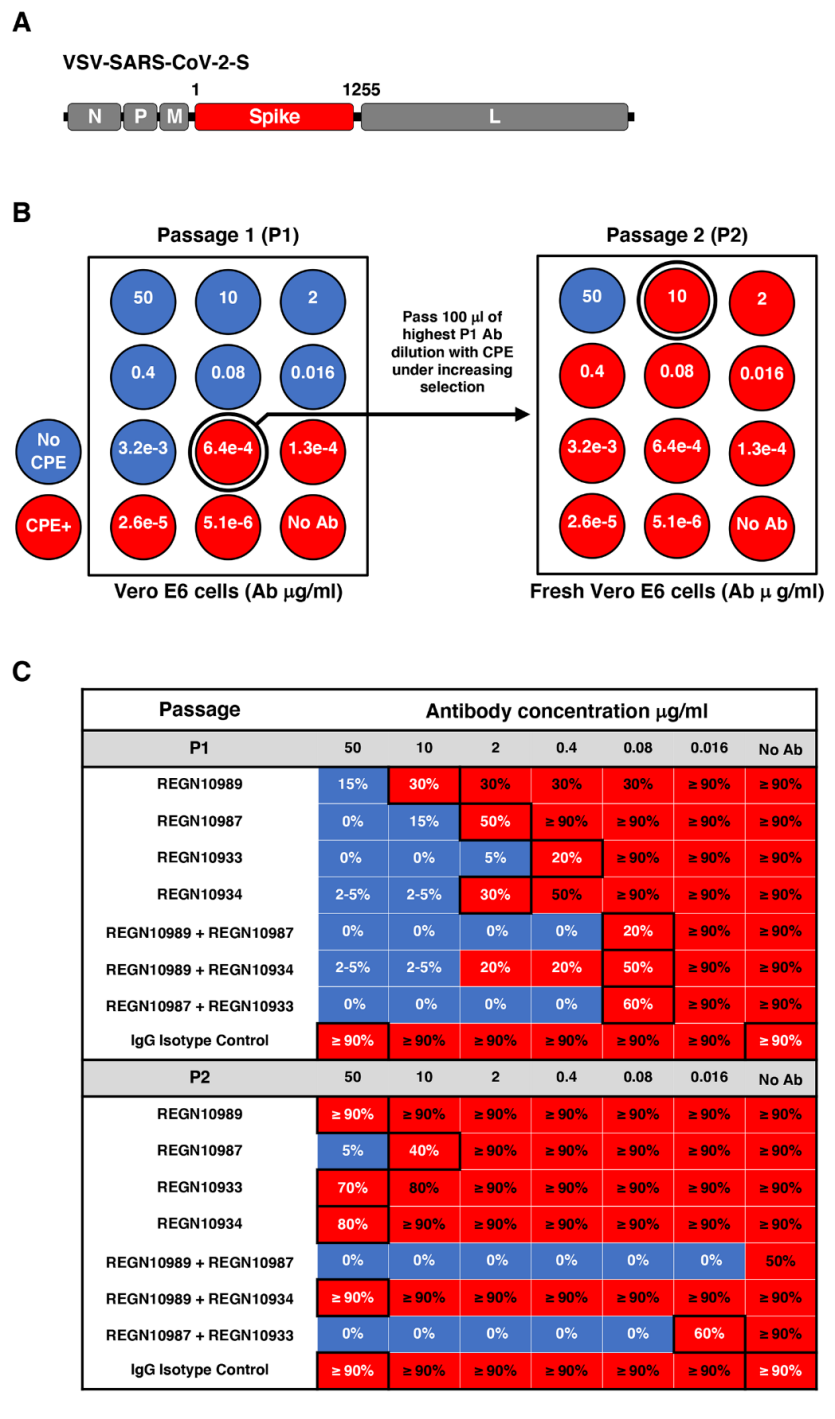
Escape mutant screening protocol. (**A**) A schematic is displayed of the VSV-SARS-CoV-2-S virus genome encoding residues 1-1255 of the spike protein in place of the VSV glycoprotein. N, nucleoprotein, P, phosphoprotein, M, matrix, and L, large polymerase. (**B**) A total of 1.5 × 10^6^ pfu of the parental VSV-SARS-CoV-2-S virus was passed in the presence of antibody dilutions for 4 days on Vero E6 cells. Cells were screened for virus replication by monitoring for virally induced cytopathic effect (CPE). Supernatants and cellular RNAs were collected from wells under the greatest antibody selection with detectable viral replication (circled wells; ≥20% CPE). For a second round of selection, 100uL of the P1 supernatant was expanded for 4 days under increasing antibody selection in fresh Vero E6 cells. RNA was collected from the well with the highest antibody concentration with detectable viral replication. The RNA was deep sequenced from both passages to determine the selection of mutations resulting in antibody escape. (**C**) The passaging results of the escape study are presented with the qualitative percentage of CPE observed in each dilution (red ≥ 20%CPE and blue < 20% CPE). Black boxes indicate dilutions that were passaged and sequenced in P1 or sequenced in P2. A no antibody control was sequenced from each passage to monitor for tissue culture adaptations.

**Fig. 2:**
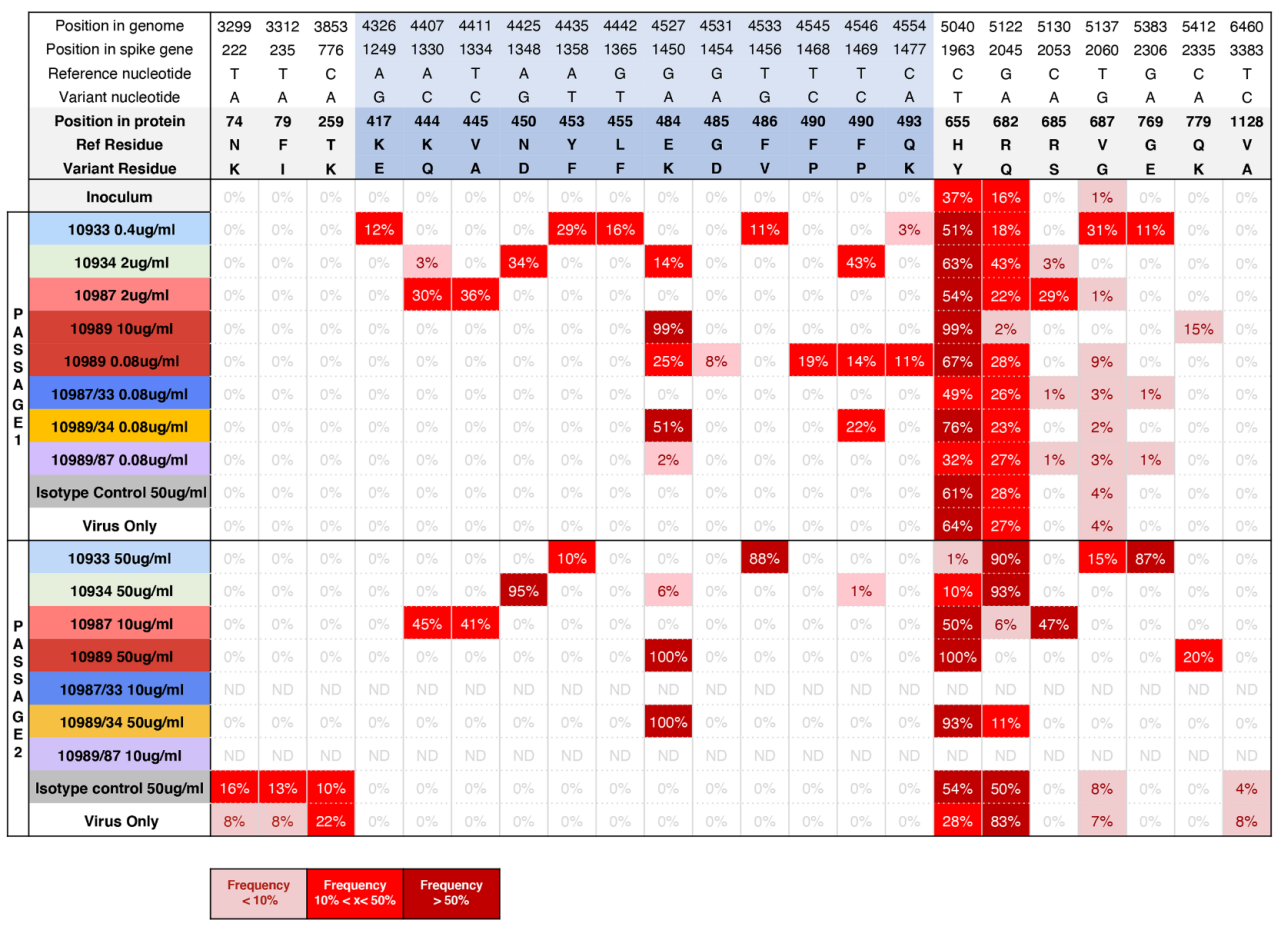
Deep sequencing of passaged virus identifies escape mutations. VSV-SARS-CoV-2-S virus was mixed with either individual or combinations of anti-spike mAbs. Viral RNA from wells with the highest mAb concentration and detectable cytopathic effect (CPE) on passage 1 or 2 (collected 4 days post-infection) was isolated and RNAseq analysis was performed to identify changes in spike protein sequence relative to input virus. For passage 2, viral RNA was isolated and sequenced from wells with high mAb concentrations (>10ug/ml) with subsequently validated escape; if no validated escape was seen at these high mAb concentrations and no virus was grown, ND is shown as no virus RNA was isolated. All mutated amino acid residues within the spike protein are shown. Specific condition (concentration in ug/ml) of the well that was selected for sequencing is shown in the left-hand column (refer to Fig. 1 for outline of the experiment). Red boxes highlight residues that were mutated relative to input virus under each condition specified in the left-hand column. Percentage in each box identifies % of sequencing reads that contained the respective mutant sequence. Residues mapping to the RBD domain are highlighted in blue.

Analysis of 22,872 publicly available unique genome sequences (through the end of May 2020) demonstrated the presence of polymorphisms analogous to two of the escape amino acid residues identified in our study, albeit at an extremely low frequency of one each. Thus, although natural variants resistant to individual antiviral antibodies were not widely observed in nature, these rare escape variants could easily be selected and amplified under the pressure of ongoing antibody treatment. Although these studies were conducted with a surrogate virus in vitro, one would expect that similar escape mutations may occur with SARS-CoV-2 virus in vivo under the selective pressure of single antibody treatment. While, the differential propensity of VSV and SARS-CoV-2 viruses to acquire mutations may impact the speed at which these escape mutants may arise, the likelihood of eventual escape remains high.

Next, we evaluated escape following treatment with our previously described antibody cocktail (REGN10987+REGN10933), rationally designed to avoid escape through inclusion of two antibodies that bind distinct and non-overlapping regions of the RBD, and which can thus simultaneously bind and block RBD function. Attempts to grow VSV-SARS-CoV-2-S virus in the presence of this antibody cocktail did not result in the outgrowth of escape mutants (Table 2, Fig. 1, B and C, and Fig. 2). Thus, this selected cocktail did not rapidly select for mutants, presumably because escape would require the unlikely occurrence of simultaneous viral mutation at two distinct genetic sites, so as to ablate binding and neutralization by both antibodies in the cocktail.

**Table 2:** Neutralization potency of individual anti-spike antibodies and antibody combinations against pseudoparticles encoding individual escape mutants-IC50 summary. Escape mutations identified by RNAseq analysis within the RDB domain were cloned and expressed on pseudoparticles to assess their impact on mAb neutralization potency. Boxes in boldface highlight conditions that resulted in at least 1.5 log decrease in IC50 relative to wild-type pseudoparticles or loss of neutralization. NC = IC50 could not be calculated due to poor neutralization ability. Reduction in IC50 less than 1 log can be seen in mAb combination conditions where one of the mAbs has no potency (ex: K444Q and REGN10933/10987). Refer to fig. S1 for full neutralization curves.

	**Anti-SARS-CoV-2 spike monoclonal antibodies**						
**Escape mutants**	**REGN10989**	**REGN10987**	**REGN10933**	**REGN10934**	**REGN10933/10987**	**REGN10989/10934**	**REGN10989/10987**
Wild-type	7.27 × 10^–12^	3.65 × 10^–11^	5.57 × 10^–11^	5.99 × 10^–11^	3.28 × 10^–11^	8.27 × 10^–12^	1.22 × 10^–11^
K417E	2.49 × 10^–11^	3.10 × 10^–11^	**8.33 × 10^–9^**	2.70 × 10^–11^	4.15 × 10^–11^	2.64 × 10^–11^	2.72 × 10^–11^
K444Q	2.47 × 10^–11^	**NC**	7.81 × 10^–11^	**5.38 × 10^–9^**	1.23 × 10^–10^	4.19 × 10^–11^	4.82 × 10^–11^
V445A	2.65 × 10^–11^	**NC**	8.82 × 10^–11^	1.42 × 10^–10^	1.54 × 10^–10^	4.08 × 10^–11^	5.74 × 10^–11^
N450D	4.10 × 10^–11^	**1.20 × 10^–9^**	7.60 × 10^–11^	**NC**	1.88 × 10^–10^	6.04 × 10^–11^	5.37 × 10^–11^
Y453F	2.77 × 10^–11^	1.04 × 10^–10^	**NC**	2.17 × 10^–10^	1.15 × 10^–10^	3.52 × 10^–11^	2.41 × 10^–11^
L455F	1.77 × 10^–11^	3.87 × 10^–11^	**NC**	4.34 × 10^–11^	5.87 × 10^–11^	1.96 × 10^–11^	1.70 × 10^–11^
E484K	**NC**	6.25 × 10^–11^	**1.13 × 10^–9^**	**NC**	6.19 × 10^–11^	**NC**	1.88 × 10^–10^
G485D	**NC**	2.34 × 10^–11^	2.05 × 10^–10^	4.47 × 10^–11^	4.71 × 10^–11^	1.19 × 10^–10^	4.58 × 10^–11^
F486V	**NC**	3.16 × 10^–11^	**NC**	3.50 × 10^–11^	8.8 × 10^–11^	1.29 × 10^–10^	6.96 × 10^–11^
F490P	**6.76 × 10^–10^**	3.75 × 10^–11^	8.65 × 10^–11^	**NC**	5.41 × 10^–11^	**2.55 × 10^–9^**	6.82 × 10^–11^
Q493K	**NC**	4.19 × 10^–11^	**NC**	3.46 × 10^–11^	3.24 × 10^–11^	**4.55 × 10^–10^**	5.94 × 10^–11^

In addition to the above cocktail, we also evaluated escape following treatment with additional combinations (REGN10989+REGN10934 and REGN10989+REGN10987), this time consisting of antibodies that completely or partially compete for binding to the RBD – i.e., two antibodies that bind to overlapping regions of the RBD. Under selective pressure of these combination treatments, there was rapid generation of escape mutants resistant to one combination, but not the other (Table 2, Fig. 1, B and C, and Fig. 2). For an antibody cocktail in which the components demonstrate complete competition (REGN10989+REGN10934), a single amino acid substitution was sufficient to ablate neutralization of the cocktail, demonstrating that both of these antibodies require binding to the E484 residue in order to neutralize SARS-CoV-2. Interestingly, such rapid escape did not occur for a different antibody cocktail in which the components exhibited only partial competition (REGN10989+REGN10987) (*3*); REGN10987 can weakly bind to RBD when REGN10989 is pre-bound. Thus even combination of antibodies that are not selected to simultaneously bind may occasionally resist escape because their epitopes only partially overlap, or because residues that would result in escape are not easily tolerated by the virus, and therefore not readily selected for.

To functionally confirm that the spike protein mutations detected by sequencing are responsible for the loss of SARS-CoV-2 neutralization by the antibodies, we generated VSV-SARS-CoV-2 spike pseudoparticles expressing the individual identified spike mutations. These pseudoparticles were used in neutralization assays with single and combination antibody treatments, and IC50 values were calculated (Table 2 and fig. S1). As expected, pseudoparticles with amino acid mutations that were selected by passaging the virus in the presence of the four single antibodies, as well as of the REGN10989+REGN10934 competing antibody cocktail, were sufficient to completely eliminate or greatly decrease the ability of these treatments to neutralize in these assays. Single escape mutants that were detected at low frequency in early passages in virus populations generated by two antibodies (e.g., K444Q by both REGN10934 and REGN10987), but were fixed in the later passage by only one of these antibodies (REGN10987), was able to ablate neutralization by both treatments. This suggests that antibodies can drive virus evolution and escape in different directions. However, if two antibodies have partially overlapping binding epitopes, then escape mutants fixed in the virus population by one can result in the loss of activity of the other – highlighting the risks of widespread use of single antibody treatments. Importantly, the REN10987+REGN10933 antibody cocktail – that consists of two antibodies that can simultaneously bind to two independent epitopes on the RBD – retains its ability to neutralize all identified mutants, even the ones that were selected for by single treatment with one of its components.

In our sequencing of passaged virus pools, we also identified multiple mutations outside of the RBD domain, most of which were present at various abundances within control samples, including the original inoculum and virus only passages (Fig. 2). The most abundant of these mutations (H655Y and R682Q) are near the S1’/S2’ cleavage site within the spike protein and contain residues within the multibasic furin-like cleavage site. Mutations and deletions in this region have been identified with tissue culture passaged VSV-SARS-CoV-2-S as well as SARS-CoV-2 viruses and likely represent tissue culture adaptations (*4*, *5*).

As RNA viruses are well known to accumulate mutations over time, a significant concern for any antiviral therapeutic is the potential for selection of treatment-induced escape mutants. A common strategy to safeguard against escape to antibody therapeutics involves selection of antibodies binding to conserved epitopes, however this strategy may not suffice. While some informed analysis can be made regarding epitope conservation based on sequence and structural analysis (*6*), the possibility of escape still exists under strong selection pressure. Indeed, escape studies performed with anti-influenza HA stem binding antibodies have shown that escape mutants can arise despite high conservation of the stem epitope between diverse influenza subtypes, with some escape mutations arising outside of the antibody epitope region (*7*, *8*). Antibodies that demonstrate broad neutralization across multiple species of coronaviruses, and thus may be targeting more conserved residues, have not been shown to be immune to escape upon selective pressure. In addition, their neutralization potency is orders of magnitude lower than that of the most potent neutralizing antibodies specific for SARS-CoV-2 (*6*, *9*–*11*). Neutralization is thought to be the key mechanism of action of anti-coronavirus spike antibodies and has previously been shown to correlate with efficacy in animal models (*12*), and may therefore prove to be the most important driver of initial clinical efficacy. However, as demonstrated with our single antibody escape studies, even highly potent neutralization does not protect against the rapid generation of viral escape mutants, and escape remains a major concern with individual antibody approaches.

The data described herein strongly support the notion that cocktail therapy may provide a powerful way to minimize mutational escape by SARS-CoV-2; in particular, our studies point to the potential value of antibody cocktails in which two antibodies were chosen so as to bind to distinct and non-overlapping regions of the viral target (in this case, the RBD of the spike protein), and thus require the unlikely occurrence of simultaneous mutations at two distinct genetic sites for viral escape. A clinical candidate selection criterion for broad potency that includes functional assessment against naturally circulating sequence variants, as well as inclusion of multiple antibodies with non-overlapping epitopes, may provide enhanced protection against loss of efficacy. Future in vivo animal and human clinical studies need to pay close attention to possible emergence of escape mutants and potential subsequent loss of drug efficacy.

## Supplementary Materials

science.sciencemag.org/cgi/content/full/science.abd0831/DC1 Materials and Methods Fig. S1 References (*13*–*19*)
